# Leptin resistance was involved in susceptibility to overweight in the striped hamster re-fed with high fat diet

**DOI:** 10.1038/s41598-017-18158-4

**Published:** 2018-01-17

**Authors:** Ying Zhao, Li-Bing Chen, Si-Si Mao, Hong-Xia Min, Jing Cao

**Affiliations:** College of Life and Environmental Science, Wenzhou University, Wenzhou, 325035 China

## Abstract

Food restriction (FR) is the most commonly used intervention to prevent the overweight. However, the lost weight is usually followed by “compensatory growth” when FR ends, resulting in overweight. The present study was aimed to examining the behavior patterns and hormones mechanisms underpinning the over-weight. Energy budget and body fat content, and several endocrine markers related to leptin signals were examined in the striped hamsters under 20% FR refed by either low-fat diet (LF group) or high-fat diet (HF group). Body mass and fat content significantly regained when FR ended, and the hamsters in HF group showed 49.1% more body fat than in LF group (*P* < 0.01). Digestive energy intake was higher by 20.1% in HF than LF group, while metabolic thermogenesis and behavior patterns did not differed between the two groups. Gene expression of leptin receptor and anorexigenic peptides of pro-opiomelanocortin and cocaine- and amphetamine-regulated transcript in hypothalamus were significantly up-regulated in LF group, but down-regulated in HF group. It suggests that effective leptin signals to the brain were involved in attenuation of hyperphagia in hamsters refed with LF. However, “leptin resistance” probably occurred in hamsters refed with HF, which impaired the control of hyperphagia, resulting in development of over-weight.

## Introduction

Food restriction has been suggested to increase the extension of life and to lose body mass of so many mammal species^1–4^. It is also the most commonly used intervention to prevent the overweight and obesity in humans^5^. However, a loss of body mass induced by food restriction is often followed by rapid recovery of the lost weight when the restriction ends, which is previously termed as “compensatory growth”^6–8^. More importantly, it has been reported that in rodents and humans compensatory growth has even been to lead to overweight, that is, the significant increases in body mass and body fat compared to that of pre-restriction^6,7,9^, indicating that this approach is rarely successful in the long-term^5,10–12^. However, in some instance some individuals showed compensatory growth following food restriction, but did not develop overweight or obesity^5,13^. On basis of energy balance, weight gain is always caused by positive energy budget indicated by elevation of energy intake and or reduction of energy expenditure.

The weight loss during food restriction is mainly the consequence of limited food intake, and the majority of the weight loss is accounted for by loss of body fat^5,7,8^. Similarly, when the food-restricted animals are allowed to be refed *ad libitum*, they overeat and may also increase the efficiency of energy intake or utilization, which contribute to compensatory growth, and even overweight^14–16^. As food intake is fixed in the restricted animals, the strategy associated with energy expenditure is the most important factor for the adaptation to food restriction^4,12,17–19^. Instead, food provided is plentiful when the restriction ends, and the hormones and neuroendocrine mechanisms related to the control of energy intake may be more significant factors defining the extent to which compensatory growth occurs^20,21^.

An anorexigenic hormone that may participate in the termination of appetitive ingestive behaviors is leptin, the product of the obesity gene (Ob) secreted primarily from adipocytes^22,23^. Leptin exerts its biological action through binding to and activating the long form of leptin receptors (LEPRb) that is extensively expressed in many brain regions, particularly in the hypothalamus^24–29^. Leptin promotes the gene expression and secretion of anorexigenic peptides of proopiomelanocortin (POMC) and cocaine-and-amphetamine-regulated-transcript (CART), but inhibit orexigenic peptides of neuropeptide Y (NPY) and agouti-related peptide (AgRp) expression in hypothalamus, and consequently inhibits appetitive ingestive behaviors^27,30^. Leptin gene expression and circulating concentrations are decreased by food restriction and increased by feeding, which is in parallel with the considerable changes in body fat^19,21,31–35^. It has been suggested that leptin serves as an important signal for the switch between fasted and fed states, allowing it to function both as a starvation and satiety signal^19,33,36,37^. The up-regulation of leptin in animals refed *ad libitum* may attenuate the hyperphagia, consequently preventing over-weight, whereas this may not be the case in the subjects showing over-weight or obesity^35^. It has been previously proposed that the failure of high circulating leptin levels in obesity to promote weight loss defines a state of so-called “leptin resistance”^24,25,27^, whereas the etiology of which remains poorly defined^38,39^. Therefore, the effectiveness of leptin signal may be a key factor associated with the compensatory growth with or without overweight.

The striped hamster (*Cricetulus barabensis*) is a major rodent in northern China and is also distributed in Russia, Mongolia, and Korea^40^. The hamsters feed on foraging crop seeds in winter, but do not store food and feed on stems and leaves of plant during summer^40^. We previously found a significant decrease in body mass and fat content in food-restricted hamsters, followed by a compensatory growth without overweight when refed *ad libitum*^41^. Exogenous leptin significantly attenuated the increase in food intake and the regaining of body mass during refeeding, indicating that it may play a crucial role in preventing overweight^35^. On the basis of the role of leptin, striped hamsters may be becoming an animal model showing resistance to overweight or obesity. However, this may not be the case, since we observed a considerable increase in body fat content in the hamsters subjected to a high-fat diet (HF)^42^. In the present study, we examined the energy budget, behavior and hormones markers in the food-restricted striped hamster followed by HF. We hypothesized that the effective leptin signal to brain might contribute to the compensatory growth without overweight, and leptin resistance resulted in overweight.

## Results

### Experiment 1

#### Body mass

**Figure 1:**
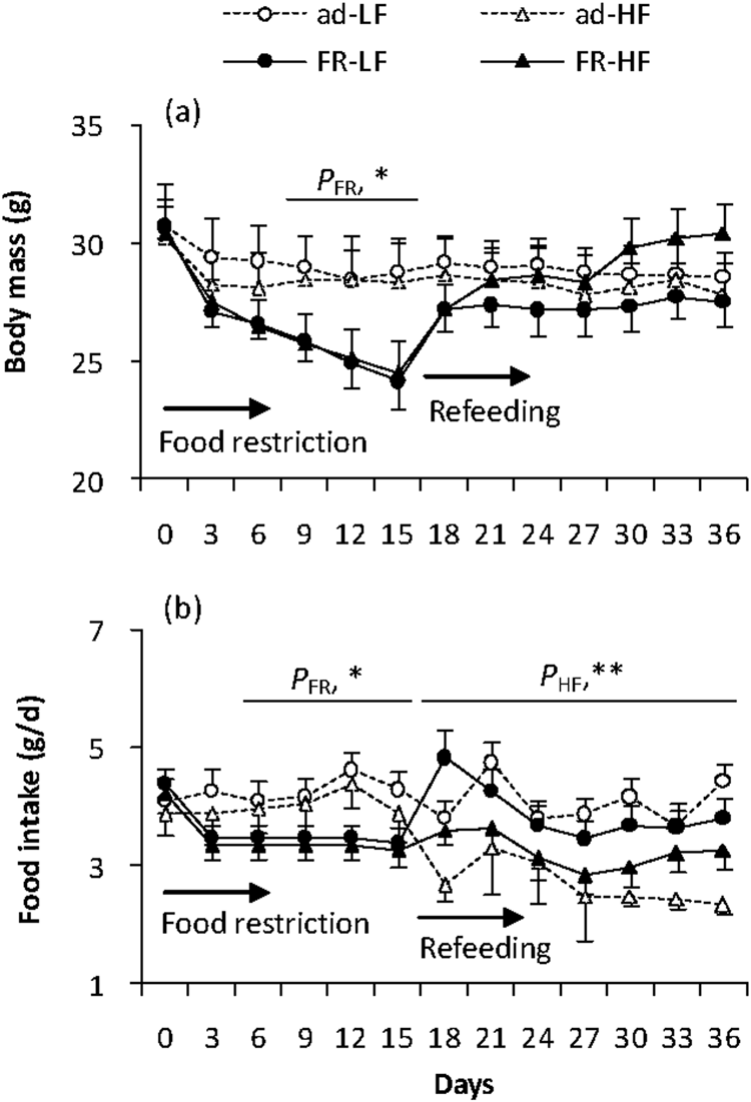
Body mass (**a**) and food intake (**b**) in striped hamsters that were restricted to 80% of *ad libitum* food intake for 2 weeks and were refed with high-fat diet for 3 weeks. Ad, animals were fed ad libitm; FR, food restriction; LF, low-fat diet; HF, high-fat diet. Data are means ± s.e.m. *P*_FR_, effect of food restriction; *P*_HF_, effect of high-fat diet; *P < 0.05; **P < 0.01.

Body mass did not differ among the four groups before food restriction started (day 0, FR, *F*_1,28_ = 0.01, *P* > 0.05, HF, *F*_1,28_ = 0.08, *P* > 0.05, Fig. 1a). Food restriction resulted in a significant decrease in body mass of FR groups, and it decreased, on average, by 20% on day 15 compared to that on day 0 (day 3 to 15, repeated measures, *F*_4,76_ = 39.40, *P* < 0.01). FR groups showed significantly lower body mass than the groups fed *ad libitum* between day 9 and 15 (day 9, *F*_1,28_ = 4.84, *P* < 0.05, day 15, *F*_1,28_ = 7.63, *P* < 0.05). The hamsters increased body mass significantly after being refed *ad libitum*, during which body mass went up by 14.0% in FR-LF group and by 24.2% in FR-HF group on day 36 compared to that on day 15 (day 15 to 36, *F*_7,133_ = 13.74, *P* < 0.01). At the end of refeeding, the hamsters in FR-HF group was heavier by 10%, on average, than the animals in other three groups, whereas the difference between the four groups was not statistically significant (day 36, FR, *F*_1,28_ = 0.35, *P* > 0.05, HF, *F*_1,28_ = 0.67, *P* > 0.05, Fig. 1a).

#### Food intake

The hamsters consumed similar food among the four groups before food restriction started (day 0, FR, *F*_1,28_ = 0.94, *P* > 0.05, HF, *F*_1,28_ = 0.38, *P* > 0.05, Fig. 1b). The food-restricted hamsters consumed significantly less food during food restriction period than those fed *ad libitum* (day 6, *F*_1,28_ = 4.12, *P* < 0.05, day 15, *F*_1,28_ = 4.99, *P* < 0.05). Food intake increased shortly after food restriction ended, and it was significantly affected by HF (day 18, *F*_1,28_ = 9.57, *P* < 0.01, day 21, day 36, *F*_1,28_ = 14.68, *P < *0.01). In detail, food intake was lower by 47.4% in ad-HF group than ad-LF group (post hoc, *P* < 0.05), while it was not different between FR-HF group and FR-LF groups (post hoc, *P* > 0.05).

#### Body fat content and serum leptin levels

**Figure 2:**
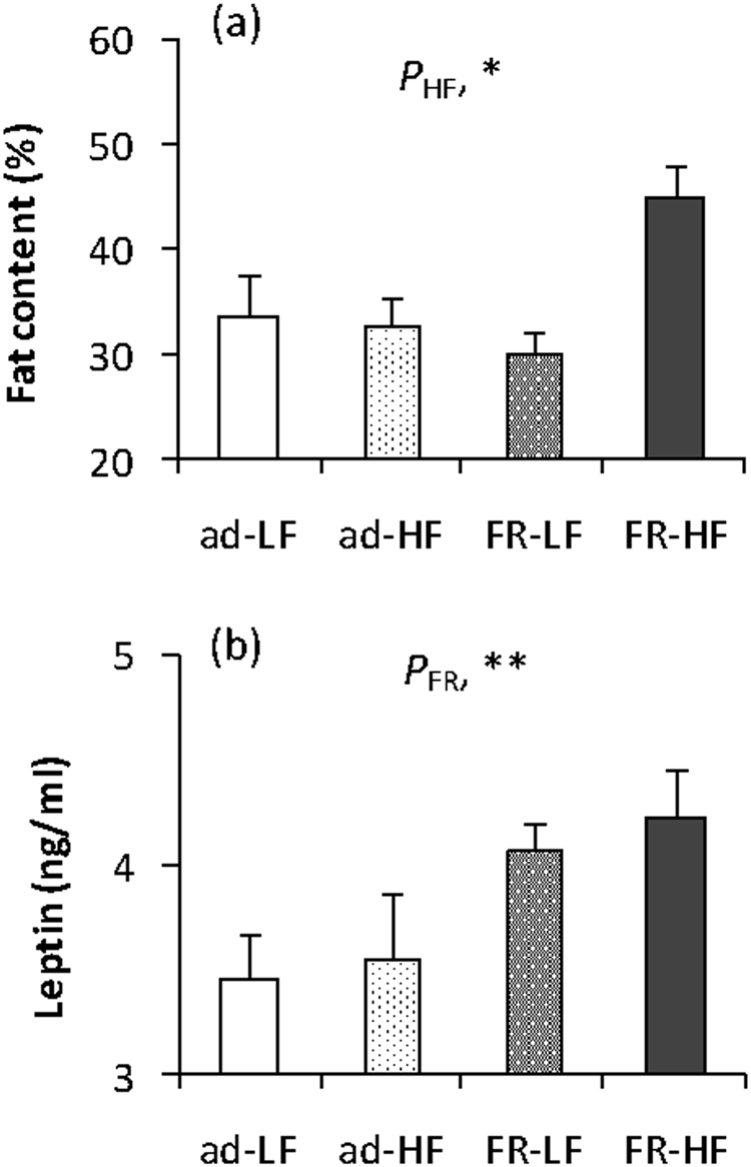
Body fat content (**a**) and serum leptin levels (**b**) in striped hamsters that were restricted to 80% of *ad libitum* food intake for 2 weeks and were refed with high-fat diet for 3 weeks. Ad, animals were fed ad libitm; FR, food restriction; LF, low-fat diet; HF, high-fat diet. Data are means ± s.e.m. *P*_FR_, effect of food restriction; *P*_HF_, effect of high-fat diet; *P < 0.05; **P < 0.01.

Body fat content was not affected by the experience of food restriction (*F*_1,28_ = 2.19, *P* > 0.05), but it was by high-fat diet (*F*_1,28_ = 5.72, *P* < 0.05, Fig. 2a). The group refed with high-fat diet showed significantly greater fat content than other three groups (*F*_1,28_ = 7.38, *P* < 0.05). Inconsistently, serum leptin levels were not affected by high fat diet (*F*_1,28_ = 0.38, *P* > 0.05), but were significantly impacted by the experience of food restriction (*F*_1,28_ = 8.34, *P* < 0.01, Fig. 2b), indicating that refeeding resulted in significant increases in serum leptin levels in the animals regardless of whether they were refed with low- or high-fat diet. In detail, leptin levels of FR-LF and FR-HF groups increased by 17.7% and 22.5%, respectively, compared to that in ad-LF groups (post hoc, *P* < 0.05).

### Experiment 2

#### Behavior

The hamsters of Re-LF groups spent similar time on feeding behavior to their counterparts of Re-HF groups, and neither Re-LF nor Re-HF group showed notable circadian rhythm of feeding behavior (supplementary materials, Fig. S1A). Both groups showed considerable circadian rhythm of activity behavior, and they spent much more time on activity during the night and less during the day. However, the activity behavior did not differ between the two groups at any time (Fig. S1B). The hamsters also showed considerable circadian rhythm of the time spent on resting behavior, but in a way opposite to the activity behavior (Fig. S1C). In detail, the resting behavior occurred mainly during the day, rather than during the night. No significant difference in resting behavior was observed between the two groups at any time over a day. It suggested that striped hamsters were active during the day, but sedate during the night regardless of the food composition. The hamsters of Re-LF and Re-HF groups showed similar accumulative time spent on feeding (*t*_22_ = 0.29, *P* > 0.05, Fig. S2A). The hamsters of Re-HF group spent 12.5% more time on activity and 9.0% less time on resting behavior than Re-LF group, whereas the difference between the two groups were not significant (activity, *t*_22_ = 1.57, *P* > 0.05, Fig. S2B; resting, *t*_22_ = 1.71, *P* > 0.05, Fig. S2C).

#### The rate of oxygen consumption

**Figure 3:**
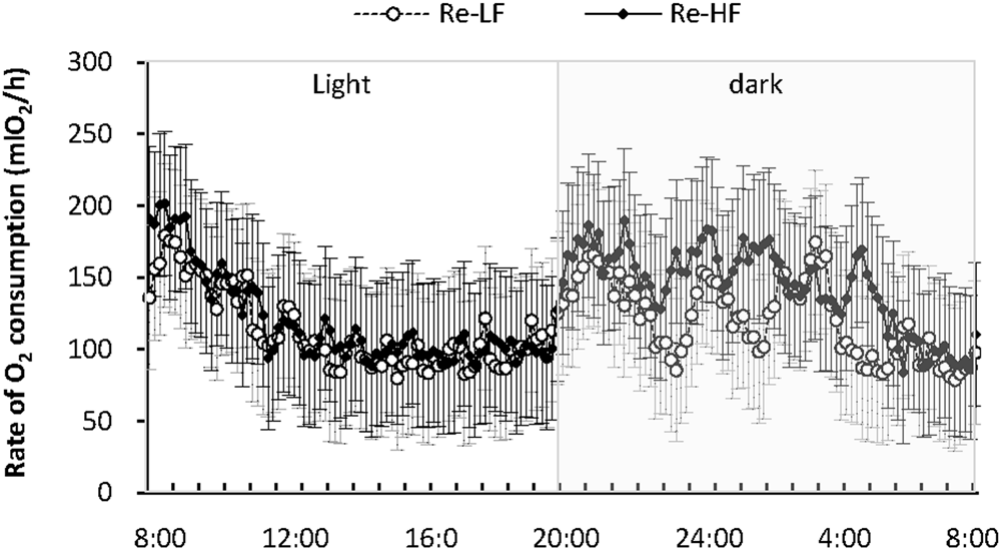
The rate of oxygen consumption over a 24 h period in striped hamsters subjected to 80% food restriction for 2 weeks followed by 2-weeks’s refeeding of low-fat diet (Re-LF) or high-fat diet (Re-HF). Data are means ± s.e.m.

The rate of oxygen consumption over a 24 h period indicated a real time measurement of energy expenditure, a rate of the summed oxygen consumption including basal metabolic rate, thermogenesis and activity. Both Re-LF and Re-HF groups showed a considerable decrease of the rate of oxygen consumption during the first several hours of the measurements (8:00–12:00), indicating an adaptation to the chamber (Fig. 3). Then the rate of oxygen consumption showed a considerable circadian rhythm, which fluctuated at low but relative stable levels during the day (12:00–8:00), and increased notably with the fluctuations at high levels. However, no significant difference was observed between the two groups at any time point (Fig. 3).

### Experiment 3

#### Body mass and food intake

**Figure 4:**
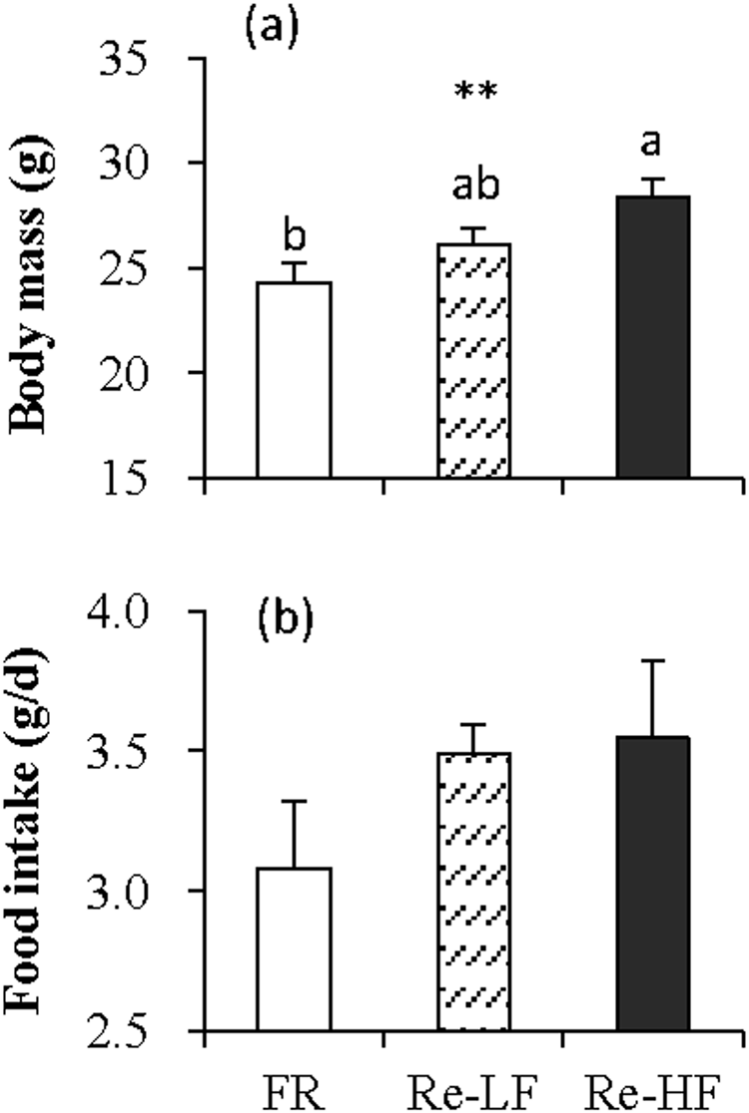
Body mass (**a**) and food intake (**b**) of striped hamsters subjected to 80% food restriction for 2 weeks (FR) followed by 1 week’s refeeding of low-fat diet (Re-LF) and high-fat diet (Re-HF). Data are means ± s.e.m. **Significant difference among the three groups (*P* < 0.01). Different letters (a or b) above the columns indicate significant difference between the groups (*P* < 0.05).

Body mass differed significantly among the three groups (*F*_2,29_ = 5.63, *P* < 0.01, Fig. 4a). Body mass averaged 24.3 ± 1.0 g in FR group, and it increased by 7.5% followed by LF refeeding (post hoc, *P* > 0.05), and by 16.8% followed by HF refeeding (post hoc, *P* < 0.05). The FR group consumed less food compared to Re-LF and Re-HF groups, but the difference among the three groups were not statistically significant (*F*_2,29_ = 1.29, *P* > 0.05, Fig. 4b).

#### Energy intake, digestibility and BMR and NST

**Figure 5:**
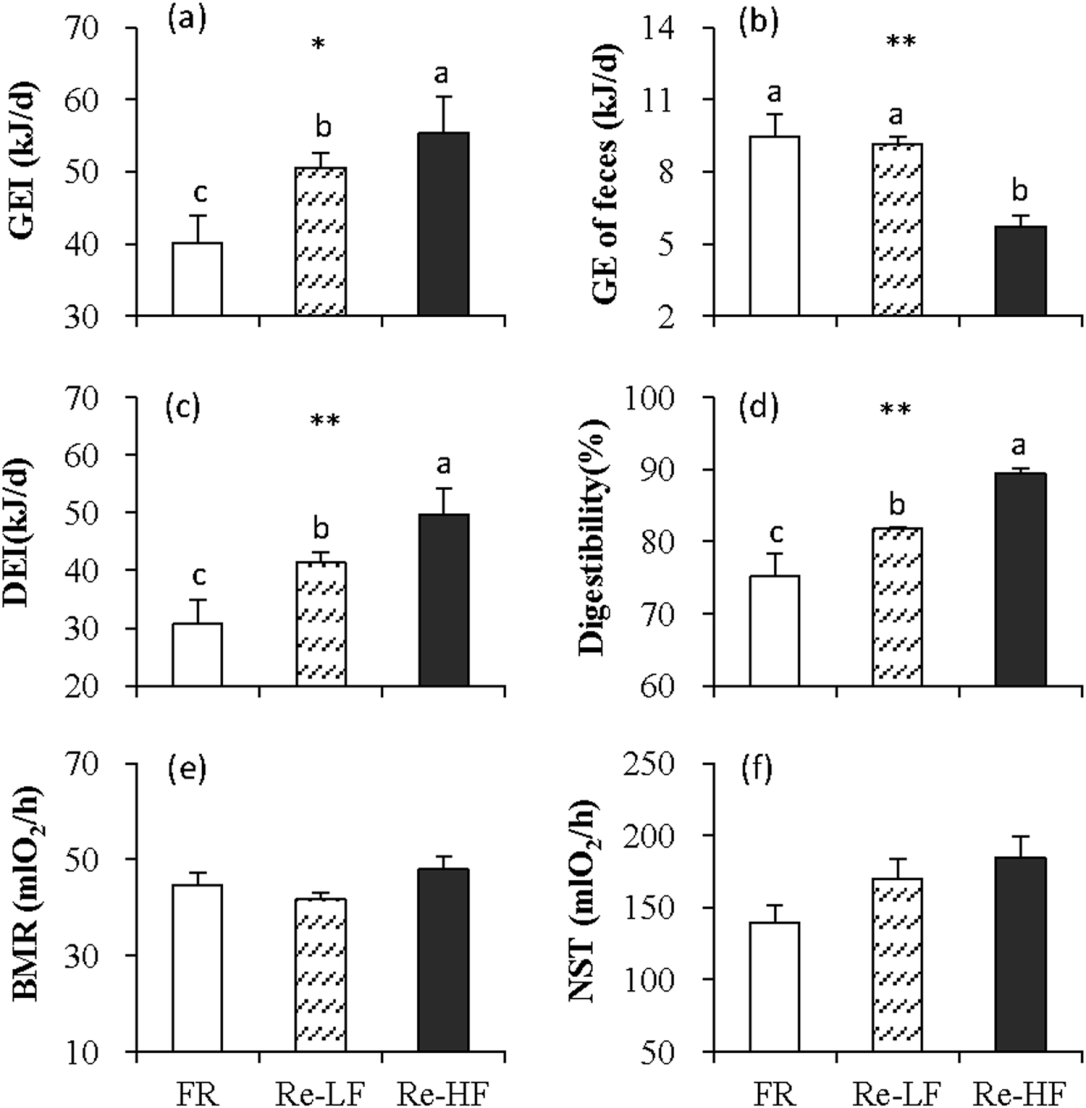
Gross energy intake (GEI, **a**), GE of feces (**b**), digestive energy intake (DEI, **c**), digestibility (**d**), basal metabolic rate (BMR, **e**) and non-shivering thermogenesis (NST, **f**) of striped hamsters subjected to 80% food restriction for 2 weeks (FR) followed by 1 week’s refeeding of low-fat diet (Re-LF) and high-fat diet (Re-HF). Data are means ± s.e.m. *Significant difference among the three groups (*P* < 0.05), **P < 0.01. Different letters (**a**,**b** or **c**) above the columns indicate significant difference between the groups (*P* < 0.05).

GEI was different among the three groups (*F*_2,29_ = 3.68, *P* < 0.05, Fig. 5a), and it was lower by 20.5% in FR group than that in Re-LF group (post hoc, *P* < 0.05). The hamsters of Re-HF group consumed 9.6% more gross energy than Re-LF group (post hoc, *P* < 0.05). The GE of feces did not differ between FR and Re-LF groups, whereas the hamsters in Re-HF group produced 37.6% less GE of feces than Re-LF group (*F*_2,29_ = 15.30, *P* < 0.01, post hoc, *P* < 0.05, Fig. 5b). Consistent with GEI, DEI differed significantly among the three groups, and it was lower in FR group, but higher in Re-HF group compared to that in Re-LF group (*F*_2,29_ = 5.65, *P* < 0.01, post hoc, *P* < 0.05, Fig. 5c). There were also significant difference in digestibility among the three groups, which decreased considerably in FR group, but increased in Re-HF group compared to that in Re-LF group (*F*_2,29_ = 21.83, *P* < 0.01, post hoc, *P* < 0.05, Fig. 5d). The three groups did not show difference in BMR (*F*_2,29_ = 1.97, *P* > 0.05, Fig. 5e) or NST (*F*_2,29_ = 2.64, *P* > 0.05, Fig. 5f).

#### Fat content and fat mass

**Figure 6:**
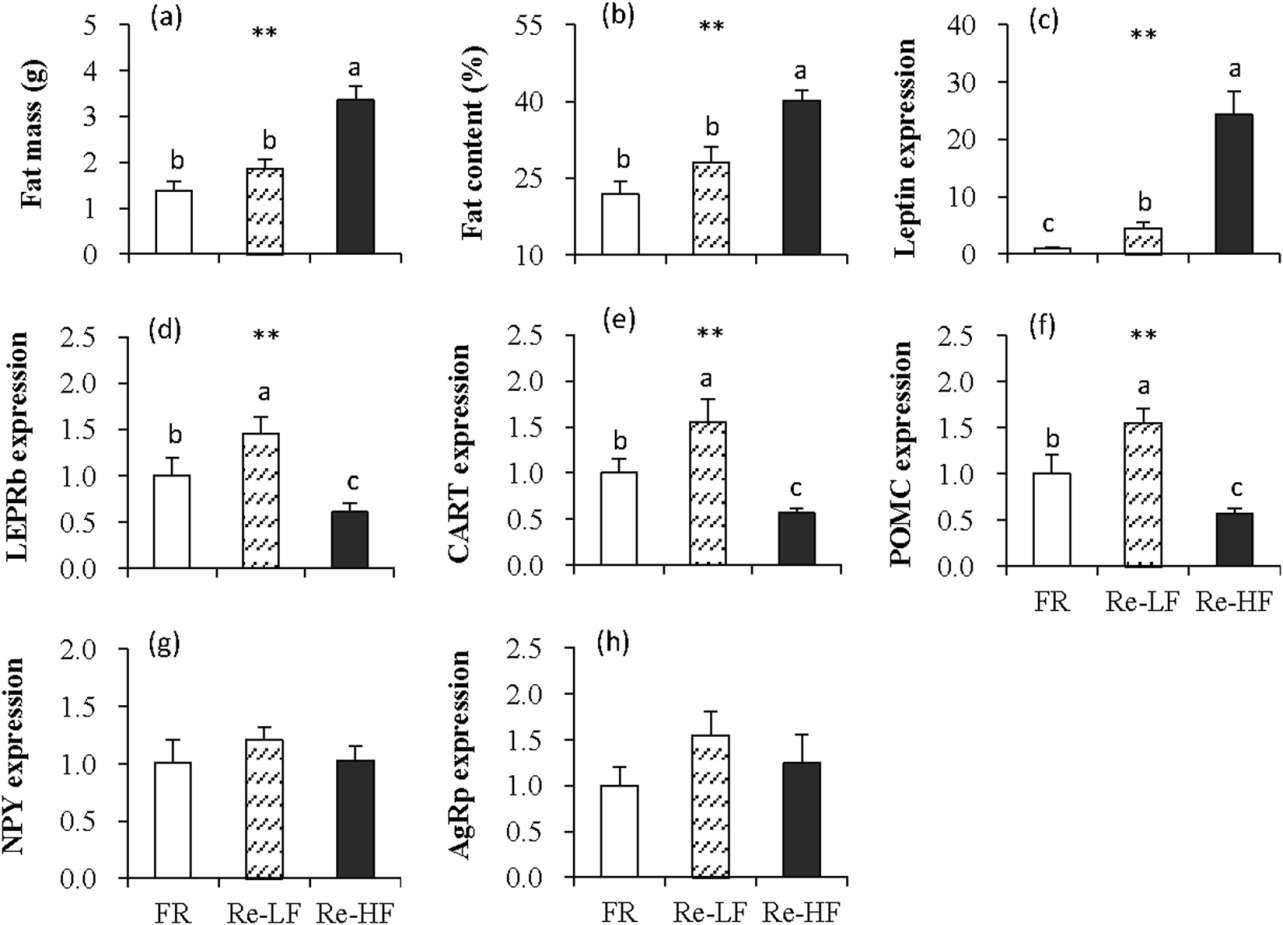
Fat mass (**a**), fat content (**b**), and leptin mRNA expression of subcutaneous fat (**c**), and mRNA expression of LEPRb (**d**), CART (**e**), POMC (**f**), NPY (**g**) and AgRp (**h**) in the hypothalamus of striped hamsters subjected to 80% food restriction for 2 weeks (FR) followed by 1 week’s refeeding of low-fat diet (Re-LF) and high-fat diet (Re-HF). Data are means ± s.e.m. **Significant difference among the three groups (*P* < 0.01). Different letters (a, b or c) above the columns indicate significant difference between the groups (*P* < 0.05).

Fat mass was significantly different among the three groups (*F*_2,29_ = 13.04, *P* < 0.01), and the Re-LF group did not differ from FR group (post hoc, *P* > 0.05), but the Re-HF group was 143.5% more fatter than FR group (post hoc, *P* < 0.05, Fig. 6a). Consistently, the Re-HF group showed significantly higher fat content than FR and Re-LF groups (*F*_2,29_ = 13.04, *P* < 0.01, post hoc, *P* < 0.05), whereas the difference between FR and Re-LF groups was not statistically significant (post hoc, *P* > 0.05, Fig. 6b).

#### Organs and serum glucose (Glu), triglycerides (TG), non-esterified fatty acid (NEFA) and insulin

Liver mass differed significantly among the three groups, and it was heavier by 24.3% in Re-HF group than that in Re-LF group (Table 1). No significant difference among the three groups was observed in other organs (Table 1). The carcass mass was significantly different among the three groups, and it increased by 19.7% in Re-HF group than that in FR group, while the difference between the FR and Re-LF group was not significant (Table 1). There was no significant difference in serum Glu concentrations among the three groups (Table 1). Neither TG nor NEFA differed significantly among the FR, Re-LF and Re-HF groups (Table 1). There was a significant difference in insulin concentrations among the three groups; that of the Re-LF and Re-HF groups was 1.5 and 10.8- fold higher than that of the FR group (Table 1).

**Table 1:** Serum Glu, TG and NEFA concentrations and, the organs mass of striped hamsters subjected to 20% food restriction and a*d libitum* refeeding.

	FR	Re-LF	Re-HF	P
Liver (g)	0.737 ± 0.076^b^	0.787 ± 0.059^b^	0.979 ± 0.069^a^	*
Heart (g)	0.143 ± 0.008	0.160 ± 0.008	0.166 ± 0.007	*ns*
Lung (g)	0.198 ± 0.007	0.221 ± 0.022	0.188 ± 0.004	*ns*
Spleen (g)	0.024 ± 0.004	0.033 ± 0.004	0.037 ± 0.003	*ns*
Kidney (g)	0.304 ± 0.019	0.323 ± 0.007	0.349 ± 0.019	*ns*
Stomach (g)	0.363 ± 0.053	0.386 ± 0.020	0.388 ± 0.018	*ns*
SI (g)	0.594 ± 0.066	0.668 ± 0.046	0.704 ± 0.029	*ns*
LI (g)	0.179 ± 0.014	0.208 ± 0.016	0.220 ± 0.015	*ns*
Caecum (g)	0.259 ± 0.058	0.230 ± 0.014	0.214 ± 0.015	*ns*
Carcass (g)	15.77 ± 0.74^b^	16.89 ± 0.54^ab^	18.88 ± 0.66^a^	**
Glu (mmol/L)	4.61 ± 0.47	4.31 ± 0.53	5.25 ± 0.29	*ns*
TG (mmol/L)	3.05 ± 0.55	2.49 ± 0.30	3.53 ± 0.63	*ns*
NEFA (µmol/L)	552.8 ± 70.8	428.1 ± 54.5	565.9 ± 87.1	*ns*
Insulin (pg/mL)	67.5 ± 9.8	166.4 ± 39.8	797.6 ± 177.8	**

Striped hamsters were subjected to 20% food restriction (FR) followed by refeeding of low-fat diet (Re-LF) and high-fat diet (Re-HF). Glu, glucose; TG, triglycerides; NEFA, non-esterified fatty acid. Data are means ± s.e.m. *, *P* < 0.05, **, *P* < 0.01; ns, non-significant (*P* > 0.05). Different letters (a and b) on the same row indicate significant difference between groups (*P* < 0.05).

#### Leptin

**Figure 7:**
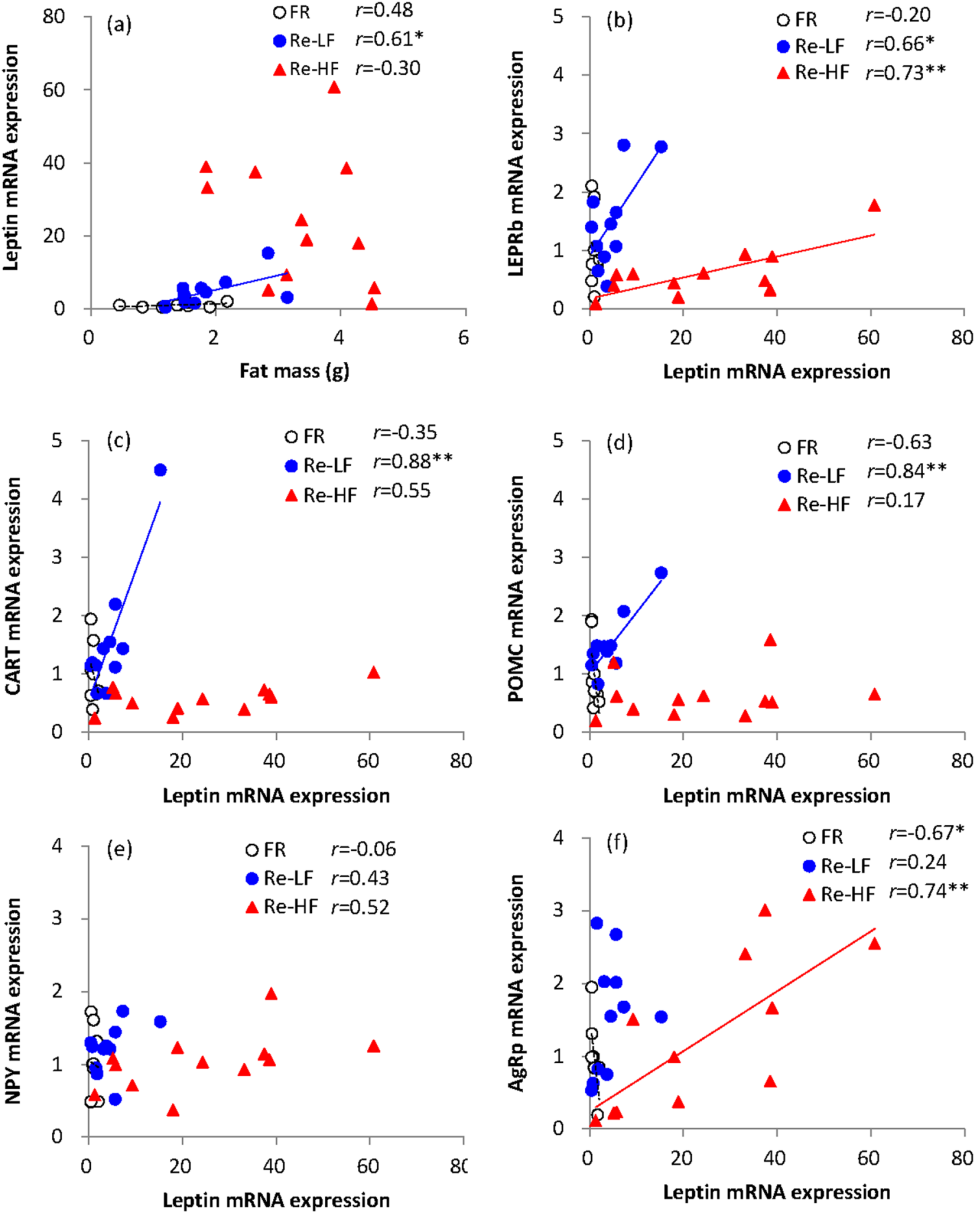
The correlations between leptin mRNA expression of fat and fat mass (**a**) and mRNA expression of LEPRb (**b**), CART (**c**), POMC (**d**), NPY (**e**) and AgRp (**f**) of the hypothalamus in striped hamsters subjected to 80% food restriction for 2 weeks (FR) followed by 1 week’s refeeding of low-fat diet (Re-LF) and high-fat diet (Re-HF).

Leptin mRNA expression of subcutaneous fat was significant different among the three group (*F*_2,29_ = 19.10, *P* < 0.01, Fig. 6c), and it was 3.5-fold up-regulated in Re-LF group compared to that in FR group (post hoc, *P* < 0.05). Leptin mRNA expression was even considerably up-regulated in Re-HF group, which was 23.3-fold and 4.4-fold, respectively, than in FR and Re-LF groups (post hoc, *P* < 0.05). There was significant positive correlation between fat mass and leptin mRNA expression in Re-LF groups (*r* = 0.61, *P* < 0.05, Fig. 7a), whereas the correlation was not observed in FR and Re-HF group (FR, *r* = 0.48, *P* > 0.05, Re-HF, *r* = 0.30, *P* > 0.05).

#### LEPRb mRNA expression of hypothalamus

LEPRb mRNA expression of hypothalamus was significantly different among the three groups, and it was up-regulated by 45.3% in Re-LF group, but was down-regulated by 39.3% in Re-HF group compared to that in FR group (*F*_2,29_ = 8.21, *P* < 0.01, post hoc, *P* < 0.05, Fig. 6d). The significant difference was also observed between Re-LF and Re-HF groups, and LEPRb expression was reduced by 58.1% in Re-HF group relative to Re-LF group (post hoc, *P* < 0.05). LEPRb expression was positively correlated with leptin expression in Re-LF (*r* = 0.66, *P* < 0.05) and Re-HF groups (*r* = 0.73, *P* < 0.01, Fig. 7b). Further, the slope of regression was much lower in Re-HF group relative to that in Re-LF group (Re-LF, y = 0.1226x + 0.8987, Re-HF, y = 0.0181x + 0.1693, *P* < 0.01), suggesting that a small increase in leptin expression might result in more notable up-regulation of LEPRb expression in Re-LF group compared to Re-HF group.

#### Neuropeptides mRNA expression of hypothalamus

CART and POMC mRNA expression differed significantly among the three groups, and both genes expression were significantly up-regulated in Re-LF group relative to FR group (CART, *F*_2,29_ = 9.37, *P* < 0.01, Fig. 6e; POMC, *F*_2,29_ = 13.43, *P* < 0.01, Fig. 6f). Re-HF group showed 63.5% and 64.9% down-regulations of CART and POMC mRNA expression than Re-LF group (CART, post hoc, *P* < 0.05; POMC, post hoc, *P* < 0.05). Leptin mRNA expression was positively correlated with CART and POMC expression in Re-LF group (CART, *r* = 0.88, *P* < 0.01, Fig. 7c; POMC, *r* = 0.84, *P* < 0.01, Fig. 7d), whereas no significant correlations were observed in FR group (CART, *r* = −0.35, *P* > 0.05, POMC, *r* = −0.63, *P* > 0.05) and Re-HF group (CART, *r* = 0.55, *P* > 0.05, POMC, *r* = 0.17, *P* > 0.05). Neither NPY nor AgRp mRNA expression were significantly different among the three groups (NPY, *F*_2,29_ = 0.63, *P* > 0.05, Fig. 6g, AgRp, *F*_2,29_ = 0.84, *P* > 0.05, Fig. 6h). There were no significant correlations between leptin and NPY expression (Fig. 7e). Leptin expression was negatively correlated with AgRp in FR group (*r* = −0.67, *P* < 0.05), but the correlation was significantly positive in Re-HF group (*r* = 0.74, *P* < 0.01, Fig. 7f).

## Discussion

The life history of many animals includes extended periods of food scarcity^18,43^. A variety of physiological and behavioral regulations are employed by the animals to cope with food shortage, and one of the most important ways is the mobilization of body fat depots^4,44,45^. Therefore, the treatment of food restriction has being used to prevent the overweight and obesity in humans^5^. Unfortunately, the weight loss is often followed by “compensatory growth” and even overweight^6–8^. In the present study, the striped hamsters showed considerable weight loss with the process of food restriction, and significant compensatory growth when food restriction ended. Interestingly, the hamsters refed with low-fat diet showed compensatory growth of both body mass and fat content, but did not develop overweight. It has been reported that laboratory mice (*Mus musculus*) regained less mass when refed with the 40% cellulose diet, a low fat diet, and did not develop obesity^5^. Similar results have been also observed in the same strain of hamsters^35^ and in other animals^46,47^. However, the hamsters refed with high-fat diet showed significantly greater content of body fat compared to the hamsters refed with low-fat diet, suggesting that the extent of fat content of diet may be an important factor defining compensatory growth or overweight. The recovery of body fat depots may be of significance for the animals to cope with the unexpected period of food shortage in the further^35,48^. Additionally, the high-fat–dense diet would induce the susceptibility to obesity following the period of food restriction.

### Effect of refeeding on body fat

High-fat diet has been well believed to be an important environmental factor promoting body fat accumulation and even obesity^49–53^ However, here we observed the body fat did not change significantly in the hamsters fed *ad libitum* with high-fat diet compared to those fed with low-fat diet. Shi *et al*. observed that the high-fat diet with 45% of fat, similar to that used in the present study, also did not affect body fat, but the high-fat diet with 60% of fat induced considerable overweight in the same strain of hamsters^42,54^. These findings suggested that the susceptibility to obesity might be associated with the fat levels of diet. More importantly, the effect of high-fat diet on body fat might be dependent on the experience of food restriction, by which the hamsters without food restriction did not develop overweight, while the hamsters experiencing food restriction did. It indicated that the experience of food shortage might also be an important environmental factor for development of obesity in the animals refed with the high-fat–dense diet. In addition, the hamsters refed *ad libitum* did not show significant changes in circulating Glu, TG and NEFA concentrations compared to the food-restricted animals, but had significantly increased insulin levels. This suggests that insulin may be involved in controlling glucose metabolism in animals subjected to high-fat diet refeeding.

### Metabolic rate and activity behavior

On basis of energy balance, regulation of energy intake or energy expenditure, or the both, might be involved in the compensatory growth or overweight following food restriction. The main energy expenditure of small mammals includes the rate of metabolic thermogenesis and the energy expended on activity^19,43,55–57^. The significant plasticity of BMR, NST and behavioral patterns has been previously reported in diversity of animals under food restriction and refeeding^4,12,17–19^. However, in the present study, we did not observe difference in BMR or NST between the hamsters refed with low- and high-fat diet. Behavioral patterns, including feeding, resting and activity behavior, also did not differ between the two groups. Inconsistent with energy expenditure, energy intake, the other side of the energy balance, was significantly different between the two groups, and it increased considerably in the hamsters refed with high-fat diet compared to those refed with low-fat diet. Overeat has been observed to contribute to compensatory growth in many other animals^14–16^. The hyperphagia was also found in striped hamsters on the first few days during low-fat diet refeeding, but it was attenuated and returned back to the baseline levels of pre-food-restriction. This might be the reason why the hamsters refed with low-fat diet showed compensatory growth, whereas did not develop obesity. Inconsistently, the hamsters refed with high-fat diet showed 20.2% greater digestive energy intake than those refed with low-fat diet, suggesting that the failure to attenuate hyperphagia might consequently result in obesity.

### Effect of *ad libitum* refeeding on leptin levels

In the present study, we also observed significant changes in serum leptin levels and leptin gene expression of subcutaneous fat depots in the striped hamsters refed with low-fat diet, which was in parallel with body fat content, but was opposite to the energy intake. Leptin gene expression and circulating concentrations have been widely reported to decrease in food-restricted animals and elevate when the restriction ends^19,21,31–35^. It has been suggested that leptin serves as a starvation signal in animals under food shortage to induce forage and/or migrate behavior^19,33,36,37,58^. Instead, the increased serum leptin levels during refeeding may function as a satiety signal, playing a role in attenuation of hyperphagia^35,44,59^. However, this might not be the case in the striped hamsters refed with high-fat diet. Here we also observed considerable increases in serum leptin levels and leptin gene expression of subcutaneous fat depots in the hamsters refed with high-fat diet. Unexpectedly, energy intake did not return the baseline levels of pre-restriction, suggesting that the up-regulated leptin failed to control hyperphagia in the hamsters refed with high-fat diet. As described previously, the failure of high leptin levels to promote weight loss defines a state of “leptin resistance”^24,25,27^. Therefore, it might indicate that the hamsters were becoming a state of “leptin resistance” under the refeeding of high-fat diet, whereas they were not after being refed with low-fat diet, providing support for the hypothesis of “leptin resistance”.

In addition, we observed that liver mass of Re-LF group did not differ from FR group, whereas that of Re-HF group was significantly greater than FR group. Both Re-HF and Re-LF showed considerable increased leptin expression, it remained uncertain why the difference occurred. Leptin has been previously demonstrated to exert tissue-specific effects in limiting fat tissue mass and lipid accumulation in liver^60^. Therefore, the possible explanation of the difference would be that leptin may have the effects in hamsters refed with low-fat diet, but “leptin resistance” may occur in hamsters refed with high-fat diet.

### Effect of *ad libitum* refeeding on hypothalamus peptides

It has been reported that leptin exerts its biological action to inhibit appetitive ingestive behaviors through binding to LEPRb in the hypothalamus, within which anorexigenic peptides of POMC and CART are likely involved^24–30^. In the present study, the hamsters refed with low-fat diet showed a significant up-regulation of LEPRb in the hypothalamus relative to food-restricted hamsters. NPY and AgRp expression did not changed, but POMC and CART were considerably up-regulated, which were consistent with the changes in leptin and LEPRb. Intraventricular injection of leptin in rats had led to significant up-regulation of gene expression of POMC and CART^61^. Therefore, it suggests that the effective leptin signals to the brain, including up-regulated LEPRb and anorexigenic peptides of POMC and CART in hypothalamus, may be probably involved in the attenuation of hyperphagia in the hamsters refed with low-fat diet.

### Leptin signaling and resistance

Leptin-mediated endocrine feedback loop between peripheral signals with the central nervous system includes three key basic components: white adipose tissue, serum leptin levels, and leptin responsive neurons in the hypothalamus^22,62–64^. Leptin exert its effect primarily by targeting LEPRb-expressing neurons in the hypothalamus. Leptin target neurons are distributed in all regions of the hypothalamus, including NPY/AgRp and CART/POMC neurons^65,66^. Defects in each component of the leptin signaling cascades are expected to result in leptin resistance. We observed that striped hamsters that were refed with high-fat diet had considerable up-regulation of leptin expression, but developed obesity compared to those fed with low-fat diet, suggesting that some components of leptin signaling may impaired. More importantly, LEPRb was significantly down-regulated in the hamsters refed high-fat diet, which accompanied by the significant increased gene expression of POMC and CART. Consistently, Mice with deletion of hypothalamic LEPRb develop early-onset obesity^67,68^. As mentioned above, LEPRb is a key components to leptin signialing, and down-regulation of LEPRb leads to a reduced responsiveness to elevated levels of endogenous leptin^29,64,69^. Thus, it suggests that the leptin resistance may occur in striped hamsters refed high-fat diet, which may impair the control of energy intake, consequently resulting in hyperphagia and development of obesity.

### In summary

The striped hamsters with the experience of 20% FR showed significant compensatory growth after being refed with low-fat diet, whereas developed considerable overweight after being refed with high-fat diet. Energy intake was significantly greater in the hamsters refed with high-fat diet compared with those refed with low-fat diet, while the energy expenditure, such as BMR, NST and behavior patterns, did not differed between the two groups. Serum leptin levels and leptin gene expression of subcutaneous fat depots significantly up-regulated during the period of refeeding compared to the period of food restriction. Gene expression of LEPRb and anorexigenic peptides of POMC and CART in hypothalamus were up-regulated under low-fat diet refeeding, but considerably down-regulated under high-fat diet refeeding. These findings suggest that the effective leptin signals to the brain might be involved in the attenuation of hyperphagia in the hamsters refed with low-fat diet, leading to a compensatory growth without over-weight. However, “leptin resistance” probably occurred in the hamsters refed with HF, which impaired the control of hyperphagia, consequently resulting in development of over-weight.

## Materials and Methods

All the procedures involving animals were reviewed and approved by the Animal Care and Use Committee of the University of Wenzhou. The methods were carried out in accordance with the approved guidelines.

### Animals and experiment protocol

Stripped hamsters were from our laboratory-breeding colony in the Animal House at Wenzhou University. Food (standard rodent chow; produced by Beijing KeAo Feed Co.) and water were provided *ad libitum* and temperature was constant at 21 ± 1 °C with a 12 L:12D photoperiod (lights on at 08: 00). Adult male hamsters, 3–4 months old, were singly housed in plastic cages (29 × 18 × 16 cm) with fresh saw dust bedding at least two weeks before the experiments.

Experimnet 1, In order to determine the effects of HF on body mass, food intake and body fat in hamsters with or without the experience of food restriction, 30 hamsters were assigned randomly into one of the following two groups: *ad libitum* feeding group (ad, *n* = 14) that were fed *ad libitum* during 5 weeks, and food restriction and refeeding group (FR-Re, *n* = 16) in which each hamster was restricted to 80% of *ad libitum* food intake for 2 weeks and refed *ad libitum* for other 3 weeks. The hamsters in each group were randomly divided into two subgroups: low fat diet group (LF), animals were fed with standard rodent chow (fat was 6.2%, carbohydrate was 35.6%, protein was 20.8%, calorific value was 17.6 KJ/g), and HF group, within which the hamsters were fed with LF diet for the first 2 weeks, and with HF diet for the other 3 weeks (HF, fat was 45.0%, carbohydrate was 35.0%, protein was 20.0%, calorific value was 19.7 KJ/g, Research Die, D12451, USA). In total, there were 4 groups: ad-LF (*n* = 7), ad-HF (*n* = 7), and FR-Re-LF (*n* = 8) and FR-Re-HF (*n* = 8).

### Body mass and food intake

Body mass and food intake were measured in a 3-days interval. Food intake was calculated from the difference between the initial food provided and the uneaten food on the next day, subtracting food residues mixed in the bedding material. The *ad libitum* food intake was measured before the experiment started, which was taken to calculate 80% of *ad libitum* food intake for each animal. The hamsters in FR groups had access to only 80% of *ad libitum* food intake during the period of food restriction, but had free access the diets throughout the refeeding period.

### Serum leptin levels

Animals were sacrificed after the experiment, and trunk blood was collected and allowed to clot for 3 hours at 4 °C. The blood was centrifuged at 4 °C (3500 g, 10 min) and serum was taken for the measurements of leptin concentrations. Serum leptin concentrations were determined by a radio-immunoassay kit (Linco Research Inc., St. Charles, MO), following the standard kit instructions. The commercial kit was valid for the striped hamster as described previously^35,44^. The inter- and intra-assay variations were 3.6% and 8.7%, respectively. The lower and upper limits of the assay kit were 1 and 50 ng/ml.

### Body fat content

After the collection of trunk blood, the gastrointestinal tracts (stomach, small and large intestine and caecum) were removed. Liver, heart, lung, spleen and kidneys were also removed. The carcass was taken, including head and tail, but excluding the organs mentioned above. The carcass was weighed (to 1 mg), and transferred into an oven, and dried to a constant mass at 60 °C. The total fat was extracted from the dried carcass by ether extraction in a Soxhlet apparatus. Fat content (%) = (total fat of carcass/dry carcass mass) × 100%^42,70^.

The experiment 2 was designed to examine the metabolic rate and behavioral patterns in the FR striped hamsters that were refed with LF or HF. Twenty five male hamsters were restricted to 80% of *ad libitum* food intake for 2 weeks, and then they were randomly assigned to two groups: Re-LF (*n* = 13) and Re-HF groups (*n* = 12), which were refed *ad libitum* with LF or HF for 3 weeks.

### Behavior

Behavioral patterns were monitored on the last three days of refeeding period using computer-connected infrared monitors (SONY, 420 TVL) and stored automatically in the computer, which was then subjected to operator analysis. As described previously, the dominant behavior of each mouse over the 24 h period was classified into one of three categories: general activity, feeding, resting and grooming behavior^41,71,72^. General activity included any active movement such as walking around the cage and climbing on the cage bars. Feeding was referred to as eating when the animal was at the hopper instead of drinking; resting was being inactive in any location of the cage^71,72^. Grooming was referred to as self-grooming. Based on personal observation, grooming behavior usually occurred shortly before and during the period of resting, which was therefore classified into the category of resting behavior. The time spent on feeding, activity and resting behavior described above was recorded using a stopwatch and expressed as means in min per hour (min/h). The accumulative time spent on each behavior over a day (min/24 h) was also calculated and presented as means ± s.e.m.

### The rate of oxygen consumption

The rate of O_2_ consumption was determined on the day next to the behavior observation, using an open-flow respirometry system (TSE, Germany). As described previously, air was pumped at a rate of 1000 mL/min through a cylindrical sealed Perspex chamber. Gases leaving the chamber were dried and sampled using an oxygen analyzer at a flow rate of 380 mL/min. the rate of O_2_ consumption was measured using a module high-speed senor (994620-CS-HSP-01). The measurements were performed throughout a whole day at 21 ± 0.5 °C, during which animals had free access to the diets. Data were collected every 1 min by a computer connected analogue-to-digital converter, and analyzed using standard software (TSE, Germany). The data was presented as mLO_2_/h, after being corrected to standard temperature and air pressure (STP) conditions.

The experiment 3 was designed to examine energy budget and fat content, and several gene expressions both in fat and hypothalamus associated with leptin and its targets genes in hamsters refed with either LF or HF. Adult male hamsters were randomly divided into one of three groups: FR, Re-LF and Re-HF groups. The FR group was restricted to 80% of *ad libitum* food intake for 2 weeks; the Re-LF and Re-HF groups were subjected to 80% FR for 2 weeks, but followed by *ad libitum* refeeding of LF and HF, respectively, for 1 week.

### Gross energy intake (GEI) and digestibility

GEI and digestible energy intake (DEI) were measured over 48 h at the end of the FR period in FR group, and of the refeeding period in Re-LF and R-HF groups. As described previously^73,74^, food was provided quantitively and the spillage of food mixed with bedding and feces were collected from each animal and separated manually after they were dried at 60 °C to constant mass. The gross energy contents of the feces were determined by IKA C2000 oxygen bomb calorimeter (IKA, Germany). GEI, digestive energy intake (DEI), GE of feces and digestibility were calculated according to the following equations^70,73,74^: GEI (kJ/d) = food take (g/d) × dry matter content of food (%) × gross energy content of food (kJ/g); GE of feces (kJ/d) = feces mass (g/d) × gross energy content of feces (kJ/g); DEI (kJ/d) = GEI − GE of feces; Digestibility (%) = (DEI/GEI) × 100%.

### Basal metabolic rate (BMR) and non-shivering thermogenesis (NST)

Both BMR and NST were measured, and quantified as the rate of oxygen consumption, using an open-flow respirometry system, as described above. Specifically, animals were fasted for 4 h before being transferred into the chamber. BMR was measured for 3 hours at 30 ± 0.5 °C (within the thermal neutral zone of this species^75,76^. The lowest rate of oxygen consumption over 10 min was taken to calculate BMR. NST as induced by subcutaneous injection of norepinephrine (NE) (Shanghai Harvest Pharmaceutical Co. Ltd.) and measured at 25 ± 0.5 °C, as described previously^76^. Two continuous stable maximal recordings were taken to calculate NST. Both BMR and NST were measured between 09:00 and 17:00 to correct for a possible effect of the circadian rhythm, and were corrected to standard temperature and air pressure (STP) conditions^77^.

### Body fat content

Animals were sacrificed on the day next to GEI measurements. Hypothalamus and subcutaneous fat depot were separated and stored at −80 °C. Liver, heart, lung, spleen and kidneys were removed and weighed (to 1 mg). The gastrointestinal tracts (stomach, small and large intestine and caecum) were also separated and weighed (to 1 mg) after contents of the gut were removed carefully. The carcass was taken, including head and tail, but excluding the brain, and weighed (to 1 mg). Carcass was then transferred in an oven, and dried to a constant mass at 60 °C, and weighed again (to 1 mg). The total fat was determined as described in experiment 1.

### Serum Glu, TG, NEFA and insulin analysis

Trunk blood was collected, and serum samples were collected as described above. Samples were stored at −80 °C until analysis. Serum Glu (Glu-F006 kit, produced by Nanjing Jiancheng Bioengineering Institute, NJBI, China), TG (TG-A110-2 kit, NJBI) and NEFA (NEFA-A402 kit, NJBI) concentrations were determined using commercial kits according to the manufacturer instructions. Levels of Glu (505 nm) and TG (505 nm) were expressed as mmol/L, and NEFA (440 nm) were presented as µmol/L. Serum insulin concentrations were determined by ELISA (Insulin mouse ELISA kit, K4271-100, Biovision, Milpitas, USA). The respective intra- and inter-assay coefficients of variations were <8% and <10%, respectively.

### Real-time RT-qPCR analysis

Total RNA was isolated from frozen subcutaneous fat and hypothalamus using the TRIzol agent (TAKARA, China). Subcutaneous adipose tissue is the main leptin producer tissue at least in other rodents including rat and mice^78–80^. In striped hamsters subcutaneous adipose tissue was considerably bigger in size than that of other sites such as visceral fat, and in food-restricted hamsters visceral fat depots were too small to be collected. As described previously^35^, mRNA expression of leptin in subcutaneous fat, and LEPRb, POMC, CART, NPY and AgRp in hypothalamus was quantified using real-time RT-qPCR methods. The qPCR was performed using Roche Light Cycler 480 real-time qPCR system (Forrentrasse CH-6343 Rotkreuz, Switzerland). All samples were quantified for relative quantity of gene expression by using actin expression as an internal standard. The gene-specific primers were presented in supplementary materials (Table S1).

### Statistics

Data were analyzed using SPSS 13.0 statistic software. Experiment 1, the changes of body mass and food intake over the period of FR-Re were analyzed using repeated measures ANOVA. The effects of FR-Re and HF on body mass, food intake, fat content and serum level were examined using two-way ANOVA. Experiment 2, the changes of behavior and O_2_ consumption rate throughout a day were analyzed using repeated measures ANOVA, and the difference between Re-LF and Re-HF groups were examined using independent-samples T test. Experiment 3, the difference among the FR, Re-LF and Re-HF groups in body mass, food intake, GEI, DEI, digestibility, body composition, body fat content and serum parameters, as well as mRNA expression of leptin and neuropeptides were examined using one-way ANOVA, followed by Tukey’s HSD post-hoc tests where appropriate. BMR and NST were analyzed using one-way ANCOVA, with body mass as a covariate. Correlations of body fat content and fat mass, and leptin mRNA expression were analyzed also using Pearson’s correlation analysis. Data were expressed as means ± s.e.m. Statistic significance was assumed at *P* < 0.05.

## Electronic supplementary material

Supplementary materials
