# Supplementary material for: Leptin resistance was involved in susceptibility to overweight in the striped hamster re-fed with high fat diet
Source: Sci Rep. 2018 Jan 17;8:920. doi: 10.1038/s41598-017-18158-4 (PMC5772526; doi:10.1038/s41598-017-18158-4)
Supplement: Supplementary file 1 — Supplementary materials [file 41598_2017_18158_MOESM1_ESM.pdf]

Leptin resistance was involved in susceptibility to overweight in the striped hamster re-fed with high fat diet

Ying Zhao[†], Li-Bing Chen[†], Si-Si Mao, Hong-Xia Min, Jing Cao[*]

College of Life and Environmental Science, Wenzhou University, Wenzhou 325035, China

Running title: Leptin resistance in striped hamster

Correspondence to:

Jing Cao Ms

College of Life and Environmental Science,

Wenzhou University,

Wenzhou, Zhejiang 325035,

People's Republic of China

Tel: +86-577-86689079

Fax: +86-577-86689257

Email: caoj@wzu.edu.cn

[†] These authors contributed equally

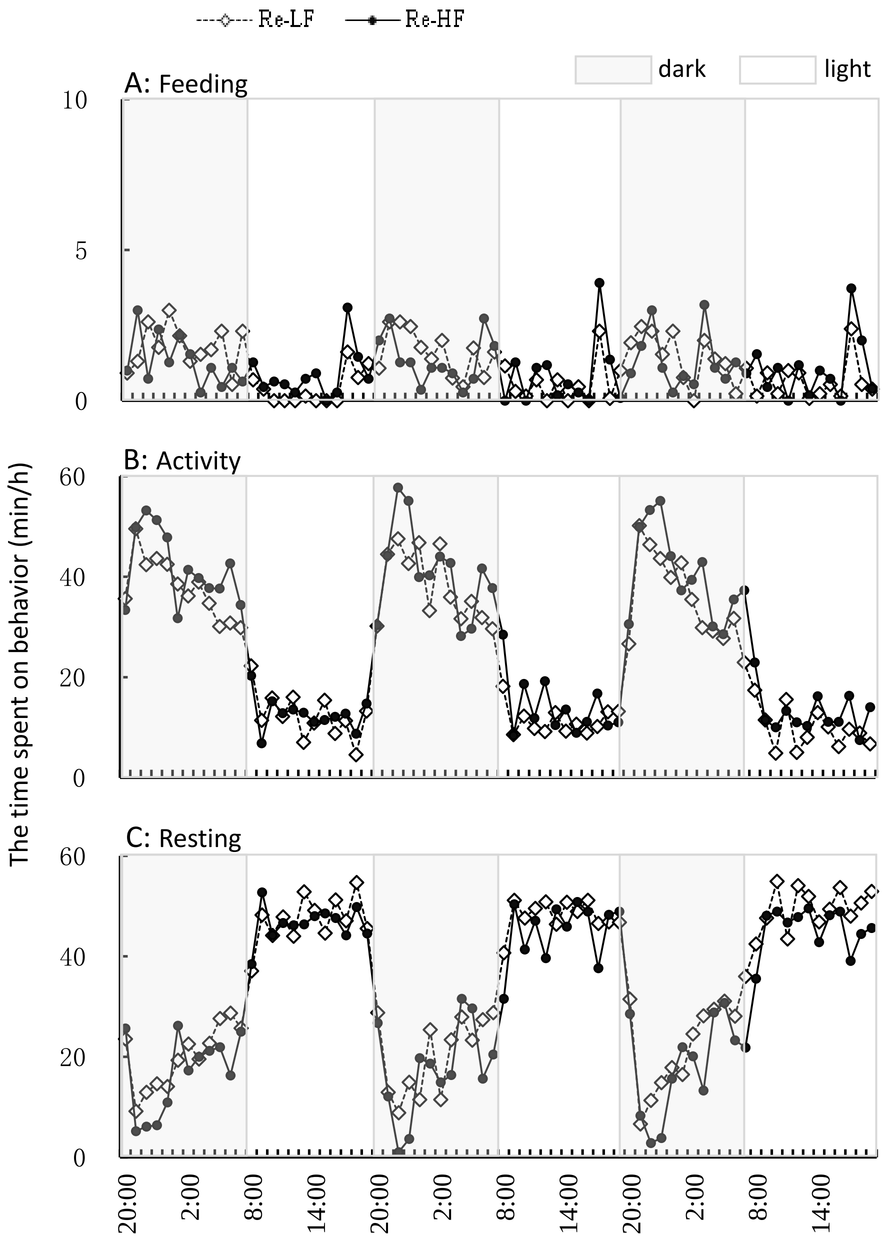

Supplementary materials, Fig. S1. The time spend on feeding (A), activity (B) and resting behavior (C) of striped hamsters subjected to 80% food restriction for 2 weeks and followed by 2-weeks' refeeding of low-fat diet (Re-LF) and high-fat diet (Re-HF). Data are means based on the hamsters from Re-LF (n=12) and Re-HF groups (n=11), respectively.

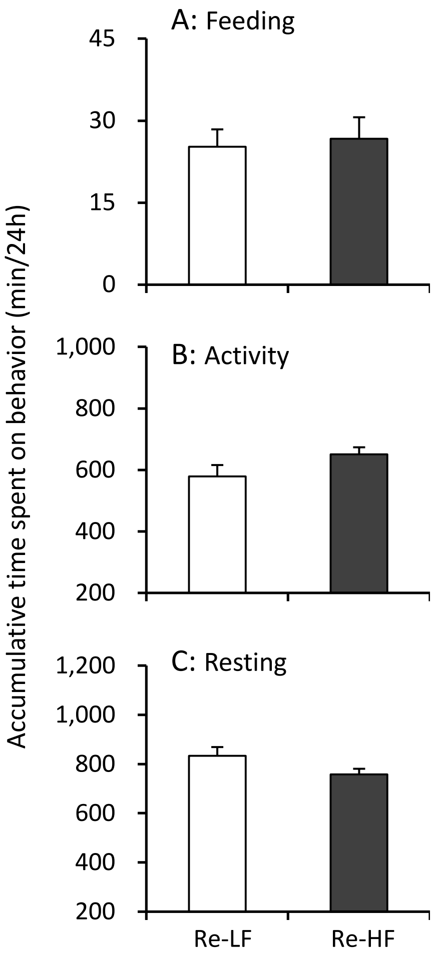

Supplementary materials, Fig. S2. The accumulative time spent on feeding (A), activity (B) and resting behavior (C) of striped hamsters subjected to 80% food restriction for 2 weeks and followed by a 2-weeks' refeeding of low-fat diet (Re-LF) and high-fat diet (Re-HF). Data are means are means ± s.e.m.

Supplementary materials, Table S1 Gene-specific primer sequences used for real-time RT-QPCR analysis

Gene	Primers	(5' to 3')
Leptin	forward	5’-AACCCTCATCAAGACCATT-3’
	reverse	5’-GCCAGCAGATGGAGAAGG-3’
POMC	forward	5’-GGTGGGCAAGAAGCGACG-3’
	reverse	5’-CTTGTCCTTGGGCGGGCT-3’
CART	forward	5’-TACCTTTGCTGGGTGCCG -3’
	reverse	5’-AAGTTCCTCGGGGACAGT-3’
NPY	forward	5’-ACCCTCGCTCTGTCCCTG-3’
	reverse	5’-AATCAGTGTCTCAGGGCTA-3’
AgRp	forward	5’-TGTTCCCAGAGTTCCCAGGTC-3’
	reverse	5’-ATTGAAGAAGCGGCAGTAGCAC-3’
LEPRb	forward	5’-CAGTGTCGATACAGCTTGGA -3’
	reverse	5’-TTGCATATTTAACTGAGGGT-3’
Actin	forward	5’-AAAGACCTCTATGCCAACA-3’
	reverse	5’-ACATCTGCTGGAAGGTGG-3’

POMC, pro-opiomelanocortin; CART, cocaine- and amphetamine-regulated transcript; NPY, neuropeptide Y; AgRp, agouti-related peptide, LEPRb, long form of the leptin receptor.
